# The Effects of Disaster on Women's Reproductive Health in Developing Countries

**DOI:** 10.5539/gjhs.v5n4p106

**Published:** 2013-04-15

**Authors:** Ronald J. Swatzyna, Vijayan K. Pillai

**Affiliations:** 1 The Tarnow Center for Self-Management, Houston, United States; 2 School of Social Work, University of Texas at Arlington, Arlington, United States

**Keywords:** reproductive health, armed conflict, disasters, developing countries

## Abstract

The objective of this study is to empirically test the effects of disasters which include natural as well as human made disasters such as armed conflict on women's reproductive health in developing countries. Data from 128 developing countries are used. It was found that average number of deaths from natural disasters and armed conflict in the East Asia and Pacific region was not significantly different from the rest of the developing world. The data are examined using structural equation analysis. This study finds that ‘armed conflict’ in developing countries presents significant reproductive health risks. The implications are discussed.

## 1. Introduction

In the last decade, women's reproductive health has become a topical area of interest with many non-governmental organizations (NGO's) particularly the United Nations, the World Health Organization, and the World Commission on Women. The International Conference on Population Development in Cairo, defined women's reproductive health as “a state of complete physical, mental, and social well-being, and not merely the absence of diseases or infirmity, in all matters relating to the reproductive system and to its functions and processes” (Pillai & Wang, 1999).

Research studies on reproductive health in developing countries have focused on reproductive health as a social as well as a public health issue. The public health approach attempts to explain the variation in reproductive health in developing countries in terms of availability of reproductive health services and the extent of knowledge about reproductive health issues among women (Mertus, 2000).

Economic inequities within and among nations have widened the gap between the very rich and very poor. As the new millennium begins, the resultant violent forces within nature and society are producing cataclysmic disasters in number, intensity, and human impact. In spite of growing instability resulting from disasters, studies on reproductive health ignore their impact. Disasters result from interactions of humans and/or nature creating a complex social event dramatically disrupting the social structure in political communities (Enarson & Morrow, 1998; Mileti et al., 1975; Dynes et al., 1987; Drabek, 1986). Furthermore, disasters are often unanticipated, unpredictable external events that influence human survival including women's control over her reproductive health. In this study, the term disaster is defined broadly to include both natural as well as socially generated events such as war and conflict. Several devastating disasters have occurred in developing countries during the last five decades (WHO, 1992).

Research on the relationship between disasters and women's reproductive health has several drawbacks. First, existing reproductive health studies have not paid attention to empirical testing of models of the impact of disasters on reproductive health (McMichael & Gifford, 2010). Current research on reproductive health lacks the theoretical as well as empirical approach toward understanding the impact of mass emergencies generated by the occurrence of disasters on reproductive health (Enarson, 2002; Fisher, 2010). Second, most studies on disasters and reproductive health focus on one category of disasters such as natural disasters and fail to account for the effect of human made disasters such as war (McGuinn, 2000). Third, when studies do consider the effects of both natural and human made disasters on reproductive health they often fall short of comparing the effects of the two at an empirical level (Joop & Jong, 2002; Cohen & Eid, 2007). Fourth, at the methodological level, prior studies have failed to adequately control for the effects of well-known influences of socio economic development on reproductive health (Usta, Farver, & Zein, 2008). In order to remedy the existing gaps in the literature on reproductive health and disasters, this study proposes to examine the net effects of natural disasters and war on reproductive health.

### 1.1 Relationship between Reproductive Health and Disasters

The five types of natural disasters considered in this study are wind storms, floods, earthquakes, hurricanes and droughts. Floods, wind storms and hurricanes account for about 30 percent of the annual direct damage while earthquakes account for another 30 percent of the total damage. There has been a dramatic increase in frequency and severity of natural disasters over the last two decades.

The impact of disasters such as natural hazards depends upon the extent of vulnerability among populations exposed to unanticipated hazardous events (Enarson & Morrow, 1998; Cutter, 1995; Blaikie et al., 1994; Downs et al., 1991; Anderson & Woodrow, 1989; Perry & Lindell, 1991; Phillips, 1993; Peacock et al., 1997). In regard to disaster, vulnerability is associated with the ability of a group to manage or withstand shock (Parasuraman & Acharya, 2000; Buchanan & Maxell, 1994; Longhurst, 1994).

In disasters, vulnerability assumes three forms based on entitlement (Parasuraman & Acharya, 2000). In developing countries, women are economically vulnerable owing to their poor labor force participation rate. As a result, women are less prepared to meet the immediate costs of recovery from the effects of disasters (Morrow & Phillips, 1999). Second, women suffer from social vulnerability owing to their low status in society. They are more likely to be neglected and discriminated against in terms of disaster assistance and aid during the recovery phase. Finally, personal vulnerability is linked to the individual's entitlement system and is dependent upon her psychological state. From a feminist perspective, the link between vulnerability and entitlement lays the foundation for understanding women's disaster vulnerability in developing countries. One consequence of high levels of vulnerability among women is that when disasters occur, women are far more exposed to microphysical dangers to women's health in developing countries than men (Enarson & Morrow, 1998).

Towards the end of the twentieth century, there was an increase in the number of internal political conflicts, refugees and displaced persons. The likelihood of wars in the developing countries has increased over time (Mehta, 1997; Chubin, 1991) owing to several social trends. The first trend is associated with a weakening of political institutions resulting in the loss of popular trust in democratically elected governments (Vorkunova, 1996). Second, during the last three decades the global market in arms has expanded exponentially. As a result, the flow of sophisticated nuclear and tactical weapons to weak states has increased (Klare, 1990). Third, growing social inequality in countries of the south is bound to increase discontent. About 80 percent of the world's population now lives in developing countries where the population is young, urban and mostly unemployed[VP1] (Head, 1989). As the gap between the rich and poor increases ethnic, religious and class conflicts are likely to emerge vastly increasing the possibility of war.

Women are affected adversely by war. They suffer poor health, and economic and social consequences of war (Busumtwi-Sam, 2002). Four immediate effects on women are poor birth outcomes, sexualized violence, displacement and trafficking. Six of the ten countries with the highest under five mortality rates in the world are war torn (Mcguinn, 2000).

Use of violence as a means to resolve conflict is a usual occurrence during war. During the course of war rape becomes symbolic of dominance and forceful entry into the body of the nation. In Rwanda, approximately 500,000 women were raped during the 1994 genocide and an estimated 5,000 pregnancies resulted from those rapes. In Sierra Leone, over 50% of women experienced some form of sexualized violence during the conflict in 1999. The National Center for PTSD Fact Sheet (2001) posits that the effects of rape on women in war are immeasurable, long lasting, and shattering to both inner and outer worlds.

During war, massive displacement of population takes place through forced migration. Displacement of population increases the spread of sexually transmitted diseases. Approximately 40 million people worldwide have been subjected to forced migration due to armed conflict and human rights violations; an estimated 80% of the displaced are women and children. Women are disproportionately affected by lack of basic services such as adequate medical care, nutrition, sanitation and shelter owing to gender based discrimination and women's powerlessness. In sum, as the intensity of conflict increases, the level of reproductive health is likely to decrease.

Modernization theory suggests that reproductive health among women is likely to improve with socio economic development (Pillai & Wang, 1999a; 1999b). Fertility rates decline due to increases in opportunity cost of women's time and decreasing dependence on children as a source of old age security (Caldwell, 1982) improves women's control over her well-being including reproductive health. Socio-economic development has a direct positive effect on reproductive health. Consequently, in order to examine the independent effects of natural disasters, and war on women's reproductive health, controls for the influences of socio-economic development becomes necessary (Bradshaw, 2004).

**Figure 1:**
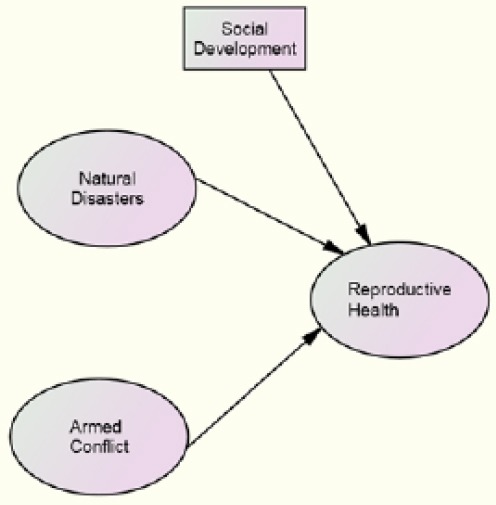
Estimated empirical model of reproductive health and armed conflict deaths

In sum, we argue that natural disasters and armed conflict decrease reproductive health. An alterative explanation is that socio-economic development increases reproductive health. Improvement in social development is likely to decrease the intensity of conflict. In order to examine the net effects of intensity of natural disasters and armed conflict on reproductive health in developing countries, we use socio- economic development as a control factor in this study. The two hypotheses tested are: as the intensity of conflict (war) as measured by number of deaths per 10,000 population, increases, reproductive health decreases. As the intensity of disasters, as measured by number of deaths per 10,000 population, increases, reproductive health decreases. Figures 1 presents the hypotheses that are tested.

## 2. Methodology

Operationalisation of natural disasters: Natural disasters include wind (hurricane, cyclone), floods, earthquakes, and drought, and, although drought does not have a sudden onset, as do the other natural disasters, it has the potential to cause widespread population displacement and societal disruption. The variable ‘natural disaster’ was compiled from occurrences of wind, earthquake, flood and drought disasters occurring in 128 developing countries inclusive of the years 1995 through 2000. This data were provided by the OFDA/CRED International Disaster Database. The reported total number affected for the six-year period was divided by the total population (2000 census) and multiplied times 10,000.

Operationalization of war (Armed conflict): The variable ‘armed conflict’ likewise, was the reported total number affected covering the same six-year period divided by the total population and multiplied times 10,000. Data are obtained from the OFDA/CRED International Disaster Database as well.

The unit of analysis is a nation state labeled as ‘developing’ by the World Bank (2000). Table 1 presents descriptive statistics on all the factors utilized in this study. The average number of people affected from natural disaster in developing countries is far greater than the mean number of those affected from armed conflict. Interestingly, in the six years studied, developing countries averaged 5.5 natural disasters affecting ten times the numbers of those affected by an average of 1.4 years of armed conflict. This preliminarily indicates that natural disaster should have a greater impact on women's reproductive health than armed conflict in developing countries just by the sheer numbers of those affected.

**Table 1:** Description of variables

Variable	Mean (Median)	Standard Deviation
URBAN	49.26 (47.01)	23.33
TFR	4.152 (4.251)	01.58
HIV	26.00 (24.01)	19.68
ATTEND	60.10 (59.51)	29.13
MAT	397.00 (225.1)	439.99
CONTRA	39.10 (37.01)	22.03
WATER	65.17 (65.01)	23.46
SANI	54.53 (55.01)	28.82
HDI	00.62 (00.62)	00.16
WARIMP	726/10,000 (726/10.000)	2051.54
NDIMP	1002/10,000 (194.82)	1610.00

Note: TFR= total fertility rate; HIV= percent of women among adults with HIV/AIDS; ATTEND= percentage of deliveries attended by skilled attendants; MAT= maternal mortality rate (per 100,000 live births); CONTRA= contraceptive prevalence rate; WATER= the percent of population with access to improved water source; SANI= percent of population with access to sanitation; HDI= Human Development Index (World Bank, 2001); NDIMP= the total of people affected by natural disaster per 10,000 population; WARIMP= the total numbers affected by armed conflict per 10,000 population.

Table 2 presents the measurement models for all the latent variables, ‘social development’ and ‘reproductive health’. The R-squared values of all the indicator variables of social development are above 30 percent and significant. All the indicators of the factors ‘reproductive health’ possess R squared values greater than 30 percent, and are significant at the .05 level. The values of the adjusted goodness of fit index are above the norm of 0.90 (Pillai & Gupta, 2000) and suggest that the proposed measurement models possess desirable levels of construct validity.

**Table 2:** Measurement model parameter estimates

Factors	Loading	R-Squared	Goodness of Fit (CFI, NFI)
Social Development			.934, .928
URBAN	.664 *	.606 *	
WATER	.852 *	.726 *	
SANI	.834 *	.696 *	
HDI	.779 *	.606 *	
Reproductive Health			.962 ,.949
TFR	.896 *	.802 *	
HIV	.548 *	.301 *	
ATTEND	.756 *	.572 *	
MAT	.748 *	.559 *	
CONTRA	.885 *	.783 *	

* Coefficient is at least twice its standard error; P < .05.

### 3. Results

Table 3 presents the standardized solution and the maximum likelihood goodness-of-fit estimates for the effects of social development on reproductive health. The direction of the effect is positive and significant. The standardized regression coefficient is .997 with a Comparative Fit Index (CFI) of .998.

**Table 3:** Structural model and goodness-of-fit estimates

Social Development Factor Loading on Reproductive Health	
	*Endogenous Factor*
*Exogenous Factor*	Reproductive Health
Social Development	.997 *
CFI, NFI	.988, .960

Note: CFI = Comparative Fit Index; NFI= Normed Fit Index;

* Coefficient is at least twice its standard error; P < .05.

Table 4 presents the standardized solutions and the maximum likelihood goodness-of-fit estimates for reproductive health and armed conflict. The hypothesis that as the intensity of conflict (war) increases reproductive health decreases is supported. The model tested is presented in Figure 1. In examining the relationship between armed conflict and reproductive health, social development is controlled. The results suggest that armed conflict has a significant negative effect net of the effect of social development. The standardized regression coefficient is -.120, significant at the .05 level. Table 4 also presents the standardized solutions and the maximum likelihood goodness-of-fit estimates for natural disasters. The model tested is very similar to the model presented in Figure 1. One difference is that the variable ‘WARIMP’ is now replaced by ‘NDIMP’ – the number of deaths from disasters per 10,000 population. The hypothesis that natural disasters have an effect on women's reproductive health finds support. Our results are not contrary to the hypothesized negative relationship between reproductive health and intensity of natural disasters. The standardized regression coefficient is -.108, significant at the .05 level. The model possesses excellent fit, as suggested by the CFI value of .967 and NFI of .932. Natural disasters appear to be associated with decreases in reproductive health levels.

**Table 4:** Structural model and goodness-of-fit estimates

Social Development and Natural Disaster Loading on RH		Social Development and Armed Conflict Loading on RH	
*Endogenous Factor*	*Endogenous Factor*	*Endogenous Factor*	*Endogenous Factor*
	Reproductive Health Standardized Coff.		Reproductive Health Standardized Coff.

Social Development	.975 *	Social Development	.973 *
Natural Disaster	-.108*	Armed Conflict	-.120 *
CFI	.967	CFI	.981
NFI	.932	NFI	.947

NOTE: CFI = Comparative Fit Index; NFI=Normed Fit Index;

* Coefficient is at least twice its standard error; P < .05.

The standardized effects in terms of the absolute values of the coefficients of armed conflict and natural disasters may be compared. The standardized coefficient for armed conflict is .120 (absolute value) and the coefficient for natural disasters is .108 (absolute value). The coefficient of armed conflict is slightly larger than the coefficient of natural disasters. Thus armed conflict has a stronger effect on reproductive health than natural disasters.

In summary, both the hypothesized models were supported. The lower the level of social development, the lower the level of reproductive health. Additionally, countries that have low levels of social development are at a greater risk for armed conflict. When comparing the effects of armed conflict and natural disaster on reproductive health, this study finds that the effect of armed conflict is larger than the effect of natural disasters. Both armed conflict and natural disasters have significant negative effects on women's reproductive health.

One approach toward examining the relationship between deaths due to natural disasters and reproductive health entails observing the distribution of deaths due to war and natural disasters across various levels of reproductive health status. In order to measure reproductive health status, a composite score of all the four indicators of reproductive health was obtained as a factor score using principal component analysis. The factor score for each country indicates the level of reproductive health based on the four indicators TFR, HIV, ATTEND and CONTRA. Lower scores indicate higher levels of reproductive health. The factor scores for all the 128 countries in our study were partitioned into three categories, 0-33 percent, 34-66 percent, 67 and above. Table 5 presents the mean number of deaths in countries due to war, and natural disasters at each of the three levels of reproductive health.

**Table 5:** Distribution of deaths due to war, and natural disasters by reproductive health status

Average Number of deaths (Per 10,000)	Levels of reproductive health		
	Low	Medium	High
War	1825	226	152
Natural Disasters	977	1129	893

The average number of deaths due to war declines steadily with increases in reproductive health. Countries with high levels of reproductive health as measured by the factor score appear to have low levels of death due to armed conflict. However, the distribution of average number of deaths due to natural disasters is not linear with reproductive health status levels. The largest average number of natural disaster deaths has occurred in countries with medium health reproductive health status. The distribution of deaths due to war, and natural deaths across reproductive health status levels suggest that the relationship between natural disaster deaths and reproductive health status has to take into account other social and economic factors related to reproductive health. The evidence of a negative relationship between reproductive health status and deaths due to armed conflict is far more persuasive than available evidence we have on the effect of natural disasters on reproductive health.

## 4. Conclusion and Discussion

To date, there has been no attention paid to the differential effect of disaster on women's reproductive health. This study makes a number of contributions. First, at the empirical level, this study develops a model of the effect of armed conflict on women's reproductive health. Second, this study contributes to the theory of disaster and disaster impact. The empirical finding that armed conflict introduces considerable decline in reproductive health has theoretical implications. This study presents empirical evidence in support of a well-known claim in the reproductive health literature. The finding that natural disasters decrease reproductive health calls for an understanding of the pathways between disasters as negative events and the responses of communities to such events (Carroll, Paveglio, Jakes, & Higgins, 2011). It is likely that in the absence of social disorganization, communities most likely come together, muster resources, and tide and overcome the negative consequences of disasters (Honigmann, 1965; Torry et al., 1979). In the course of recovering from the consequences of disasters, community members may help each other in a myriad of different ways so that community members may make gains, including in reproductive health

This study has implications for organizations funding and providing disaster assistance. The first decade of the millennium has begun with a global upsurge of disasters including armed conflict. Understandably, disaster relief organizations must have access to the most current research involving disasters’ impact for the purpose of deciding where it is most equitable to place money and resources. In many developing countries, disaster mitigation and management is designed and implemented to reduce financial losses as well as the loss of human lives. Our results suggest that it is important to address the issue of armed conflict as a factor in the decline of reproductive health in developing countries. As helpless victims of war and conflict, women bear the short term as well long term ill effects of conflict. It is essential to extend the role of existing disaster management agencies in the developing countries to plan for the ill effects of war on women's reproductive health.

Natural disasters and war bring about similar consequences such as loss of life, property and population displacement. Many effective techniques of disaster mitigation and disaster management have been developed. These techniques may be extended to address the disastrous effects of war. The consequences of natural disasters such as hurricanes and winds are often reduced in the presence of a social network of friends and relatives who often aid the disaster victims in coping with the disaster's deleterious effects. However, when armed conflict occurs, vast disruptions brought upon the community often reduce the effectiveness of social network systems to successfully mitigate the effects of disruption. Therefore, the reduction of vulnerable populations is key to reducing the impact of natural disasters and armed conflicts.

These, however, will necessitate broader participation of women in decision-making positions to address women's specific concerns. Perhaps the first step in the reduction of this vulnerability involves women's involvement in the process that leads to armed conflict. Women play only a marginal role in the processes that generate armed conflict. Yet, women bear the high costs of war. This is reflected in the declining level of reproductive health resulting from armed conflict. A social empowerment approach provides a broad strategy within which they may acquire resources and procedural means within which they can enhance their own lives. More essentially, they should be part of peacekeeping missions and all peacekeepers should be trained and sensitized to the special needs of women.

Findings from this study may be generalized taking into account several methodological limitations. The first limitation concerns the measurement of variables in our model of reproductive health and disasters. Armed conflict was measured using only one indicator, the number of war-related deaths per thousand populations. Data on health risks resulting from war should be incorporated in developing a scale for the measurement of war related consequences. Furthermore, the number of people dead during war may far exceed the number reported owing to political reasons. For these reasons, it is necessary to develop a scale of intensity of armed conflict using a number of social, political and economic indicators. Second, the tests of hypothesized causal links are at best correlational. Further replication is necessary to confirm the preliminary evidence of the causal relationship between disasters and reproductive health presented in this study.
